# Identification of protein-coding and non-coding RNA expression profiles in CD34^+ ^and in stromal cells in refractory anemia with ringed sideroblasts

**DOI:** 10.1186/1755-8794-3-30

**Published:** 2010-07-15

**Authors:** Mariana O Baratti, Yuri B Moreira, Fabiola Traina, Fernando F Costa, Sergio Verjovski-Almeida, Sara T Olalla-Saad

**Affiliations:** 1 Department of Internal Medicine, School of Medical Science, Hematology and Hemotherapy Center, University of Campinas, 13083-970 Campinas, SP, Brazil; 2 Departmento de Bioquimica, Instituto de Quimica, Universidade de São Paulo, 05508-900 São Paulo, SP, Brazil

## Abstract

**Background:**

Myelodysplastic syndromes (MDS) are a group of clonal hematological disorders characterized by ineffective hematopoiesis with morphological evidence of marrow cell dysplasia resulting in peripheral blood cytopenia. Microarray technology has permitted a refined high-throughput mapping of the transcriptional activity in the human genome. Non-coding RNAs (ncRNAs) transcribed from intronic regions of genes are involved in a number of processes related to post-transcriptional control of gene expression, and in the regulation of exon-skipping and intron retention. Characterization of ncRNAs in progenitor cells and stromal cells of MDS patients could be strategic for understanding gene expression regulation in this disease.

**Methods:**

In this study, gene expression profiles of CD34^+ ^cells of 4 patients with MDS of refractory anemia with ringed sideroblasts (RARS) subgroup and stromal cells of 3 patients with MDS-RARS were compared with healthy individuals using 44 k combined intron-exon oligoarrays, which included probes for exons of protein-coding genes, and for non-coding RNAs transcribed from intronic regions in either the sense or antisense strands. Real-time RT-PCR was performed to confirm the expression levels of selected transcripts.

**Results:**

In CD34^+ ^cells of MDS-RARS patients, 216 genes were significantly differentially expressed (q-value ≤ 0.01) in comparison to healthy individuals, of which 65 (30%) were non-coding transcripts. In stromal cells of MDS-RARS, 12 genes were significantly differentially expressed (q-value ≤ 0.05) in comparison to healthy individuals, of which 3 (25%) were non-coding transcripts.

**Conclusions:**

These results demonstrated, for the first time, the differential ncRNA expression profile between MDS-RARS and healthy individuals, in CD34^+ ^cells and stromal cells, suggesting that ncRNAs may play an important role during the development of myelodysplastic syndromes.

## Background

Myelodysplastic syndromes (MDS) are a heterogeneous group of clonal hematological disorders characterized by ineffective hematopoiesis with morphological evidence of marrow cell dysplasia resulting in peripheral blood cytopenia [1,2]. Low-risk MDS are characterized by profound anemia and transfusion dependency, and a relatively low risk of progression to acute myeloid leukemia. Refractory anemia with ringed sideroblasts (RARS) is a subtype of low-risk MDS in which an excess of iron accumulates in the perinuclear mitochondria of ringed sideroblasts in the form of mitochondrial ferritin (MtF) [3-5]. However, the molecular genetic basis of RARS remains unknown.

Gene expression profile of hematopoietic progenitor cells of MDS patients demonstrates the involvement of genes related to differentiation and proliferation of progenitor cells [6-9]. Furthermore, there is increasing evidence that, in certain hematological disorders, the marrow microenvironment is abnormal, both in composition and function [10]. In MDS, the adherent layer of bone marrow stroma is defective in supporting normal myelopoiesis *in vitro*, presenting a poor maintenance of hematopoietic stem cells [11]. Alteration of stroma components can be implicated in the modification of the development and apoptosis of hematopoietic cells [12,13].

The ENCODE project identified and characterized the transcriptionally active regions in 1% of the human genome, and described that the majority (63%) of transcripts was of long non-coding RNAs (ncRNAs). These transcripts resided outside GENCODE annotations, both in intronic (40.9%) and intergenic (22.6%) regions [14]. Non-coding RNAs are known to be involved in different biological processes such as cell survival and regulation of cell-cycle progression [15], transcriptional or post-transcriptional control of gene expression [16,17], genomic imprinting [18] and biogenesis of mature RNAs through changes in the intron-exon structure of host genes [19-21]. In addition, introns have been shown to be sources of short ncRNAs such as microRNAs [22] and small nucleolar RNAs [23].

Microarray technology has permitted a refined high-throughput mapping of the transcriptional activity in the human genome [24], and has revealed different expression signatures of long intronic ncRNAs in prostate, liver and kidney [25]. In addition, sets of long intronic ncRNAs were found to be responsive to physiological stimuli such as retinoic acid [26] or androgen hormone [27]. Long ncRNAs change the interpretation of the functional basis of many diseases [28,29]such as α-thalassemia [30], Prader-Willi syndrome [31], and cancer [32-35]. Recently, a myelopoiesis-associated regulatory intergenic non-coding RNA transcript has been described [36].

In the present study, expression profiles of CD34^+ ^and stromal cells of MDS-RARS patients and healthy individuals were characterized with a 44 k combined intron-exon oligoarray platform, allowing the identification of protein-coding and intronic ncRNA expression signatures in MDS-RARS patients.

## Methods

### Patients

Bone marrow (BM) samples were collected from 4 healthy subjects and 7 MDS patients, seen at the Hematology and Hemotherapy Center, University of Campinas. All patients were diagnosed as RARS according to the French-American-British (FAB) classification and did not present chromosomal abnormalities; they received no growth factors or any further MDS treatment. Patients' characteristics are shown in Table 1. All patients and healthy subjects provided informed written consent and the National Ethical Committee Board of School of Medical Science - University of Campinas approved the study.

**Table 1:** Characteristics of MDS-RARS patients at the time of BM collection.

Patients (n)	Gene expression analyses for CD34^+ ^cells	Gene expression analyses for stromal cells	Age (years)	Gender	Hb (g/dL)	ANC (×10^3^/uL)	Platelet count (×10^3^/uL)	BM blasts (%)	RS (%)
1	Array	None	72	F	7.2	0.95	75	2	30
2	Array, qPCR	None	78	M	8.3	1.23	136	0	58
3	Array, qPCR	qPCR	62	M	12.4	0.99	110	2	16
4	Array, qPCR	Array, qPCR	70	M	10.2	5.34	322	0	38
5	qPCR	Array, qPCR	69	F	6.7	0.51	72	0	22
6	qPCR	Array, qPCR	56	M	8.4	2.81	754	0	91
7	None	qPCR	75	M	6.3	0.75	216	0	27

Hb indicates hemoglobin; ANC, absolute neutrophil account; BM, bone marrow; and RS, ring sideroblasts

### CD34+ cell and stromal cell selection

BM mononuclear cells were isolated by density-gradient centrifugation through Ficoll-Paque Plus (GE Healthcare, Uppsala, Sweden), labeled with CD34 MicroBeads, and CD34^+ ^cells were isolated using MACS magnetic cell separation columns (Miltenyi Biotec, Mönchengladbach, Germany) according to the manufacturer's instructions. The purity of CD34^+ ^cells was at least 92% as determined by fluorescence-activated cell sorting (FACS), using anti-CD34 antibody (Caltag Laboratories, Burlingame, CA). The mononuclear cells without CD34^+^, were plated onto Iscove's Dulbeccos (IMDM) (Sigma, St Louis, MO, USA) supplemented with 10% fetal bovine serum and 10% horse serum. Supernatant with non-adherent cells was removed weekly and replaced with fresh medium. When the monolayer was established (90% confluence) cells were trypsinized and plated under the same conditions. After three re-platings, a homogeneous cell population was obtained and the stromal cells were evaluated by FACS for the absence of CD34, CD45 and CD68 antigens.

### RNA extraction

Total RNA was extracted with RNAspin Mini RNA Isolation Kit (GE Healthcare, Freiburg, Germany). The integrity of RNA was evaluated using Agilent 2100 Electrophoresis Bioanalyzer (Agilent Technologies, Santa Clara, CA).

### Microarray experiments and data analysis

Gene expression measurements were performed using 44 k intron-exon oligoarrays that were custom-designed by the group of Verjovski-Almeida and collaborators [25] following Agilent Technologies probe specifications [37] and were printed by Agilent. The array comprises 60-mer oligonucleotide probes for 24,448 long (>500 nt) ncRNAs mapping to 6,282 unique gene loci, with genomic coordinates of the Human Genome May 2004 Assembly (hg17). Non-coding RNAs probed on the array are transcribed from either intronic regions in both the sense or the antisense strands with respect to a protein-coding gene [25] or from 1,124 intergenic regions [38]. The array also comprises 13,220 probes for the respective protein-coding genes [25]. Gene locus name annotation for intronic ncRNA is that of the protein-coding gene of the same locus; intergenic ncRNA is annotated with the name of the nearest protein-coding gene in that chromosome. All annotations were updated as of December 2009. The array design is deposited in the GEO platform under accession number GPL9193.

For each individual sample, 150 ng total RNA was amplified and labeled with Cy3 or Cy5 using the Agilent Low RNA Input Fluorescent Linear Amplification Kit PLUS, two-Color (Agilent Technologies) according to the manufacturer's recommendations. Labeled cRNA was hybridized using Gene Expression Hybridization Kit (Agilent).

Slides were washed and processed according to the Agilent Two-Color Microarray-Based Gene Expression Analysis protocol (Version 5.5) and scanned on a GenePix 4000 B scanner (Molecular Devices, Sunnyvale, CA, USA). Fluorescence intensities were extracted using Feature Extraction (FE) software (version 9.0; Agilent). A gene was considered expressed if probe intensity was significantly (p > 0.05) higher than the local background intensity, as calculated by the FE software. The software then applied local background subtraction and corrected unequal dye incorporation using the default LOWESS (locally weighted linear regression) method. We have included into further statistical analyses only those genes that were detected as expressed in all samples.

Data were normalized among the samples by quantile [39] using Spotfire DecisionSite^® ^for Microarray Analysis (TIBCO Software Inc, Somerville, MA, USA). Genes differentially expressed between MDS-RARS and healthy individuals were identified with the Significance Analysis of Microarray (SAM) statistical approach [40] followed by a patient leave-one-out cross validation [41], which consisted in removing one sample and determining a new set of significantly altered genes using the remaining samples. This procedure was repeated for each sample, computing the statistical significance of each gene in the various leave-one-out datasets. For both CD34^+ ^and stromal cell samples we used the following parameters: two-class unpaired responses, t-statistic, 500 permutations. We considered as significantly altered those genes that showed a minimum fold change of 1.7 and maximum false discovery rate (FDR) of 1% for CD34^+ ^or 5% for stromal cells among all leave-one-out datasets. Less stringent parameters (5% FDR for CD34^+ ^or 15% for stromal cells) were used for generating a list of altered genes that was uploaded to Ingenuity Pathways Analysis (IPA) software (Ingenuity^® ^Systems, http://www.ingenuity.com) for identification of relevant altered gene networks. The software assigns statistical scores, taking into account the user's set of significant genes, network size, and the total number of molecules in Ingenuity Knowledge Base. The network score is the negative logarithm of p-value, which reflects the probability of finding the focus molecules in a given network by random chance. The identified network is then presented as a graph, indicating the molecular relationships between gene products. The raw data has been deposited in Gene Expression Omnibus (GEO) http://www.ncbi.nlm.nih.gov/geo/ database under accession number GSE18911.

Gene Ontology Annotation (GOA) database http://www.ebi.ac.uk/GOA/ was used for annotating the biological processes of proteins encoded by transcripts that were statistically significantly altered and showed at least 1.7-fold change in expression levels. Intronic non-coding RNA transcripts were annotated according to the corresponding protein-coding genes transcribed from the same *loci*. Functional descriptions of the genes were obtained from the Online Mendelian Inheritance in Man (OMIM) database of the National Center for Biotechnology Information (NCBI) http://www.ncbi.nlm.nih.gov/.

### Quantitative real-time RT-PCR

Real-time RT-PCR was performed to confirm expression levels of expression data for selected transcripts. Reverse transcription was performed using Superscript™III Reverse Transcriptase (Invitrogen Life Technologies). Primers (Table 2) were designed using PRIMER3 software (version 0.4.0) http://frodo.wi.mit.edu/primer3/ with published sequence data from the NCBI database. For real-time PCR analysis, Power SYBR^® ^Green PCR Master Mix (Applied Biosystems) was used, according to the manufacturer's instructions, and the reactions were run in a 7500 Real-Time PCR Systems (Applied Biosystems) for 40 cycles. Each sample measurement was performed in triplicate and a negative control, "No Template Control", was included for each primer pair. Expression of transcripts was normalized to the HPRT endogenous gene, and the relative expression was calculated as 2^-ΔΔCT^, where ΔΔCT is the C_T _value difference for each patient normalized by the average C_T _difference of samples from healthy subjects (ΔΔCT method) [42].

**Table 2:** Sequences of primers for real-time RT-PCR assays.

	sense	antisense
		
Genes selected for validation in CD34^+ ^cells		
*MY5OC*	5'CGTGGCAGAAGAGGCATACA3'	5'AGCGAGCCGACACTGTCTTT3'
*GCDH_ncRNA*	5'aaggagctttgggtttttgt3'	5'cgccttagtgacagtctccag3'
*HCK*	5'GAGTTCATGGCCAAAGGAAG3'	5'GGAGGTCTCGGTGGATGTAG3'
*PPIF_ncRNA*	5'atcgagctttgggggtagat3'	5'agcaacagtgtagcgcaatg3'
*NR4A2*	5'ACTCCAACCCGGCTATGAC3'	5'CATGGAGCCAGTCAGGAGAT3'
*NR4A2_ncRNA*	5'cttccgggtgtctgagaaag3'	5'tttgcatgtgctaggagctg3'
*NR4A3*	5'TCCAGATACTGTCCCACTGACC3'	5'TCTGGATACATCAATGGAGGCT3'
*NR4A3_ncRNA*	5'ccgttaagcacctggttttc3'	5'aggcgctgagaattattgga3'
		
**Genes selected for validation in stromal cells**		

*SPINT2*	5'TCTGTTTCTCTGGGAGGTAGGA3'	5'CGATCAGCGAGGAAACAACT3'
*HLA-E*	5'ATCGTGGGCATCATTGCT3'	5'GAGTAGCTCCCTCCTTTTCCA3'
*SEMA3A*	5'AGGGACCGAAAACAACGTC3'	5'CGTGGGTCCTCCTGTTTCTA3'
*TBCD_ncRNA*	5'TCTTGCCTGGTGAGTGTGAG3'	5'AAGGTGTTGGAAGGGAGGAG3'
*SOLH_ncRNA*	5'AGTCTCCAGAGTGGCATGTG3'	5'AAAGGTAGGCAGGGGAGGTA3'
		
**Endogenous gene**		

*HPRT*	5'TCCAGCAGGTCAGCAAAGAA3'	5'GAACGTCTTGCTCGAGATGT3'

## Results

### Protein-coding and non-coding transcripts expression profiles in CD34^+ ^cells

**Figure 1:**
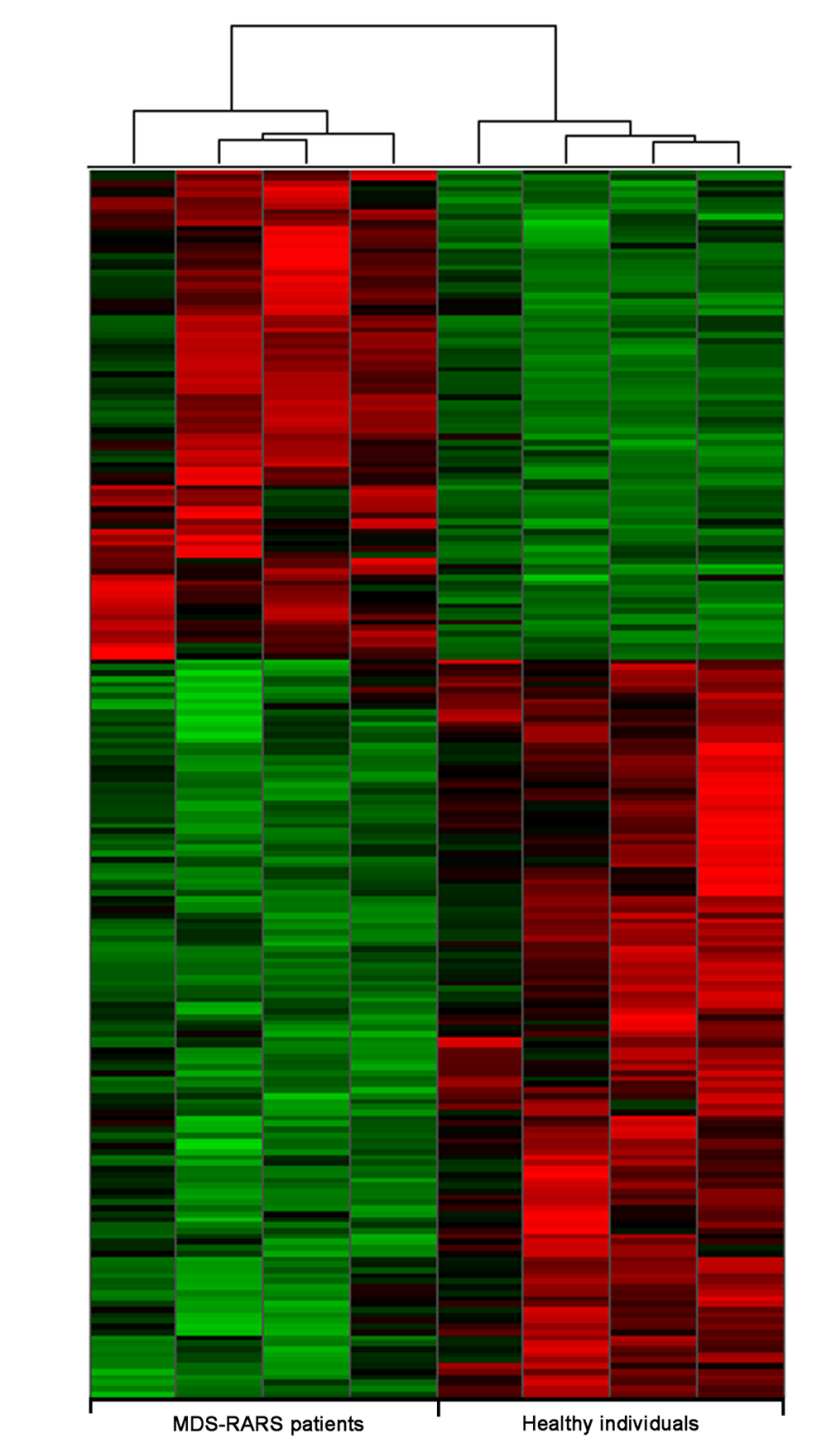
**Differentially expressed transcripts in CD34^+ ^cells of MDS-RARS patients and healthy individuals**. The panel shows the expression matrix of 216 significantly differentially expressed genes in CD34^+ ^cells of MDS-RARS patients compared to healthy individuals (SAM FDR <1% and fold change ≥1.7). Each row represents a single gene probe (151 protein-coding and 65 ncRNAs) and each column represents a separate CD34^+ ^donor sample. Donor samples were clustered according to the correlation of expression profiles using the Unweighted Pair-Group Method, which resulted in two homogenous groups: MDS-RARS patients (4 columns at left) and healthy individuals (4 columns at right). Expression level of each gene is represented by the number of standard deviations above (red) or below (green) the average value for that gene across all samples. In MDS-RARS patients, a total of 87 genes were up-regulated and 129 down-regulated.

CD34^+ ^cells obtained from 4 patients with MDS-RARS (nos. 1-4; Table 1) were compared with CD34^+ ^cells of healthy individuals using custom-designed combined intron-exon expression oligoarrays [25]. The oligoarray includes probes for protein-coding genes and for both sense and antisense strands of ncRNAs, as described in the Methods. In order to reduce the effect of individual variability, thus promoting the identification of a robust gene expression signature of MDS-RARS, Significance Analysis of Microarrays (SAM) [40] was combined with a patient leave-one-out cross validation [41]. A total of 216 significantly (q-value ≤ 0.01) differentially expressed transcripts between MDS-RARS patients and healthy individuals were identified (Figure 1), being 129 down-regulated and 87 up-regulated in MDS-RARS (Additional file 1). Interestingly, 65 differentially expressed transcripts were ncRNAs, 32 down-regulated and 33 up-regulated (Table 3).

**Table 3:** ncRNAs with significantly altered CD34^+ ^expression in MDS-RARS patients in relation to healthy individuals.

Gene Locus name^1^	Locus ID	ncRNA Probe Coordinates	Probe Strand	Type	Orientation relative to protein coding gene	q value^2^	Fold Change
**Down-regulation in MDS-RARS**							
*NEIL1*	79661	chr15:73434282-73434341	+	Intronic	Sense	0.000	-4.45
*BLNK*	29760	chr10:97961400-97961459	-	Intronic	Sense	0.000	-3.59
*KLHL5*	51088	chr4:38941503-38941562	+	Intronic	Sense	0.004	-2.92
*LEF1*	51176	chr4:109454925-109454984	+	Intergenic	Antisense	0.001	-2.80
*GABPB1*	2553	chr15:48447272-48447331	+	Intergenic	Antisense	0.000	-2.55
*TLE1*	7088	chr9:81478431-81478490	-	Intronic	Sense	0.000	-2.30
*TNFAIP3*	7128	chr6:138230463-138230522	-	Intronic	Antisense	0.000	-2.21
*IGSF10*	285313	chr3:152637104-152637163	+	Intergenic	Sense	0.000	-2.19
*CPSF6*	11052	chr12:67954327-67954386	-	Intronic	Antisense	0.000	-2.16
*MORN1*	79906	chr1:2326963-2327014	-	Intronic	Sense	0.003	-2.15
*ASAP2*	8853	chr2:9299234-9299293	+	Intronic	Sense	0.003	-2.14
*ST7OT1*	93653	chr7:116186492-116186551	-	Intergenic	Sense	0.004	-2.13
*TLE1*	7088	chrX:64411696-64411755	+	Intronic	Sense	0.000	-2.12
*NASP*	4678	chr1:45744001-45744060	+	Intronic	Sense	0.001	-2.10
*TLE1*	7088	chr9:81478491-81478550	-	Intronic	Sense	0.000	-2.10
*PIAS2*	9063	chr18:42643244-42643303	-	Intergenic	Sense	0.000	-2.05
*NUP153*	9972	chr6:17815444-17815503	+	Intergenic	Sense	0.001	-2.04
*ZNF76*	7629	chr6:35364312-35364371	+	Intronic	Sense	0.002	-2.02
*ERO1LB*	56605	chr1:232705155-232705214	-	Intronic	Antisense	0.000	-1.98
*CPSF6*	11052	chr12:67954327-67954386	-	Intronic	Antisense	0.000	-1.97
*ASXL1*	171023	chr20:30410722-30410781	+	Intronic	Sense	0.001	-1.94
*C5orf13*	9315	chr5:111094583-111094642	+	Intronic	Antisense	0.000	-1.86
*PRG4*	10216	chr1:183013558-183013617	-	Intronic	Antisense	0.000	-1.86
*GABPB1*	2553	chr15:48448783-48448842	+	Intergenic	Antisense	0.003	-1.85
*AFF1*	4299	chr4:88268668-88268724	+	Intronic	Sense	0.000	-1.83
*PAG1*	55824	chr8:82068816-82068875	-	Intronic	Sense	0.001	-1.83
*LOC441242*	441242	chr7:63977853-63977912	-	Intergenic	Sense	0.003	-1.82
*AASS*	10157	chr7:121332812-121332871	+	Intronic	Antisense	0.001	-1.79
*GCDH*	2639	chr19:12871038-12871097	-	Intronic	Antisense	0.000	-1.79
*SNORA71B*	26776	chr20:36482692-36482751	-	Intergenic	Sense	0.001	-1.77
*AFF3*	3899	chr2:99698165-99698216	-	Intronic	Sense	0.004	-1.76
*RBM4B*	83759	chr11:66192338-66192397	+	Intronic	Sense	0.001	-1.73
**Up-regulation in MDS-RARS**							
*NR4A3*	8013	chr9:99668743-99668802	+	Intronic	Sense	0.001	5.52
*NR4A2*	4929	chr2:157011025-157011084	-	Intronic	Sense	0.002	4.49
*CCDC146*	57639	chr7:76467266-76467325	-	Intronic	Antisense	0.003	4.03
*MARCH1*.	55016	chr4:164805948-164806007	-	Intergenic	Sense	0.002	3.77
*THBS1*	7057	chr15:37668391-37668450	+	Intronic	Sense	0.001	2.77
*FN1*	2335	chr2:216086310-216086369	+	Intronic	Antisense	0.000	2.71
*RALGPS1*	9649	chr9:126945696-126945747	+	Intronic	Antisense	0.000	2.70
*SERPINA1*	5265	chr14:93919199-93919258	+	Intronic	Antisense	0.001	2.51
*EHBP1L1*	254102	chr11:65104384-65104443	-	Intronic	Antisense	0.001	2.38
*PPIF*	10105	chr10:80781110-80781169	-	Intronic	Antisense	0.003	2.36
*ANK3*	288	chr10:61625808-61625867	-	Intronic	Sense	0.004	2.30
*PPIF*	10105	chr10:80781110-80781169	+	Intronic	Sense	0.001	2.29
*ZSCAN10*	84891	chr16:3082319-3082378	-	Intronic	Sense	0.004	2.24
*B3GNT5*	84002	chr3:184455731-184455790	+	Intronic	Sense	0.001	2.21
*CYBASC3*	220002	chr11:60883741-60883792	-	Intronic	Sense	0.002	2.21
*NR4A2*	4929	chr2:157012210-157012269	+	Intronic	Antisense	0.004	2.15
*IFI30*	10437	chr19:18147121-18147180	-	Intronic	Antisense	0.004	2.12
*DHX38*	9785	chr16:70696571-70696630	+	Intronic	Sense	0.000	2.11
*FAM50A*	9130	chrX:153324988-153325047	+	Intronic	Antisense	0.002	2.05
*SESTD1*	91404	chr2:179802621-179802672	+	Intronic	Antisense	0.000	1.91
*CC2D1A*	54862	chr19:13891701-13891760	-	Intronic	Antisense	0.002	1.88
*SNX20*	124460	chr16:49264159-49264218	-	Intronic	Sense	0.000	1.88
*ICA1*	3382	chr7:7932382-7932433	-	Intronic	Sense	0.000	1.86
*NCOA3*	8202	chr20:45714557-45714616	-	Intronic	Antisense	0.003	1.86
*FRAS1*	80144	chr4:79383367-79383426	+	Intronic	Sense	0.004	1.85
*TMBIM6*	7009	chr12:48437217-48437268	+	Intronic	Sense	0.001	1.85
*CD44*	960	chr11:35184235-35184294	-	Intronic	Antisense	0.000	1.83
*PPP1R15A*	23645	chr19:54068439-54068498	-	Intronic	Antisense	0.002	1.82
*PKM2*	5315	chr15:70288031-70288090	+	Intronic	Antisense	0.000	1.81
*RP5-1022P6.2*	56261	chr20:5491968-5492027	-	Intronic	Sense	0.002	1.81
*DAGLBETA*	221955	chr7:6274325-6274384	+	Intronic	Antisense	0.003	1.78
*RAD51L1*	5890	chr14:67951712-67951763	-	Intronic	Antisense	0.001	1.77
*S100A4*	6275	chr1:150330268-150330327	+	Intronic	Antisense	0.001	1.71

^1^Gene locus name for intronic ncRNA is that of the protein-coding gene of the same locus; intergenic ncRNA is annotated with the name of the nearest protein-coding gene in that chromosome.

^2^Minimum significance among all patient Leave-one-out analyses

Differentially expressed protein-coding genes were related to cell adhesion, apoptosis, ion transport and regulation of transcription (Table 4). Six protein-coding genes, namely *ABCB7*, *EBF1*, *IFI30*, *IL10RA*, *NR4A2 *and *VEGF*, have been previously shown in the literature as differentially expressed in MDS-RARS [7,43-45]. In addition, we identified a number of protein-coding transcripts not previously described as altered in MDS-RARS.

**Table 4:** Biological processes of genes differentially expressed in CD34^+ ^and stromal cells from MDS-RARS patients.

	Genes Differentially Expressed	
	
Biological Process^1^	in CD34+ Cells	in Stromal Cells
apoptosis	↓*CYFIP2*, ↓*MGC29506*, ↑*PHLDA2*, ↑*SGK1*, ↓*SOCS2*, ↓*TNFRSF21*, ↑*TNFRSF1B*, ↓*UNC13B*	___________
blood coagulation	↓*SEC63*, ↑*SERPINA1*	___________
cell adhesion	↓*AEBP1*, ↓*COL5A1*, ↓*CYFIP2*, ↑ITGAX, ↓*JAM2*, ↓*LAMB2*, ↓*LAMC1*, ↓*PCDH9*, ↓*PDZD2*, ↓*ROBO1*, ↑*VCAN*	↑*RGMB*
cell cycle	↓*E2F7*, ↓*NASP*, ↓*UHRF1*	____________
cell differentiation	↓*NAV1*, ↓*ROBO1*	↑*SEMA3A*
cell motility	____________	↓*SPINT2*
cell proliferation	↓*CD81*, ↓*DLG3*, ↓*FLT3*, ↑*LRP1*, ↓*NASP*, ↓*NPY*, ↑*S100A11*, ↓*UHRF1*, ↑*VEGFA*	____________
DNA replication	____________	↑*KRT7*
exocytosis	↓*NKD2*, ↓*RIMS3*, ↓*UNC13B*	____________
immune response	↓*CD19*, ↑*CTSS*, ↓*ERAP1*, ↑*OAS1*, ↓*PXDN*	↓*HLA*-E
inflammatory response transport	↑*AOAH*, ↓*C5*, ↓*BLNK*, ↑*LY96*	____________
	↓*ABCB7*, ↓*COL5A1*, ↑*HVCN1*, ↓*KCNE1L*, ↓*KCNMB3*, ↓*KCNMB4*, ↓*NKD2*, ↓*NPY*, ↑*SGK1*, ↑*SGSH*, ↑*SLC8A1*, ↑*SLC11A2*, ↓*SLC12A2*, ↑*SLC37A2*, ↓*SLC39A8*, ↑*SLC43A2*	↑*SCFD2*, ↓GRIA3
oxidation reduction	↓*AASS*, ↑*CYB5R1*, ↑*IFI30*, ↓*PTGR1*, ↓*PXDN*	↑*LEPREL1*
protein amino acid phosphorylation	↓*FLT3*, ↑*HCK*, ↑*SGK1*	↓TNIK
regulation of transcription	↓*ECHDC2*, ↓*AEBP1*, ↓*AFF3*, ↓*BACH2*, ↓*CEP290*, ↓*E2F7*, ↓*EBF1*, ↓*ELP2*, ↑*ETV3*, ↑*JAZF1*, ↓*LOC400713*, ↑*NCOA4*, ↑*NR4A2*, ↑*NR4A3*, ↓*POU2AF1*, ↑*RXRA*, ↓*TLE1*, ↓*UHRF1*, ↓*ZNF711*, ↓*ZNF91*	___________
rRNA processing	____________	___________
signal transduction	↓*ANK3*, ↑*ARHGEF10L*, ↓*C13orf18*,↑*NR4A2*, ↓*PSD3*, ↑*S100A11*, ↓*TLE1*, ↓*TNFRSF21*	____________
others	↑*AADACL1*, ↓*AADAT*, ↑*ACPP*, ↓*ACSM3*, ↑*ADCY7*, ↓*AHI1*, ↓*ALS2CR4*, ↓*AUTS2*, ↓*C16orf67*, ↓*C1orf21*, ↓*C9orf58*, ↑*CALML4*, ↓*CCDC136*, ↓*CLIP3*, ↑*COLT1*, ↑*CTSH*, ↓*CYYR1*, ↑*DDX3X*, ↑*DDX3Y*, ↓*DPY19L2*, ↓*EBF1*, ↑*EHBP1L1*, ↓*FAAH*, ↑*FGD6*, ↑*FGFR1OP2*, ↓*HHAT*, ↓*HS3ST1*, ↑*IL10RA*, ↑*JHDM1D*, ↓*LAYN*, ↑*LFNG*, ↓*LRIG1*, ↓*MMP11*, ↓*MPDZ*, ↓*MYO1D*, ↓*MYO5C*, ↓*NEIL1*, ↓*PLS3*, ↓*SH2D4B*, ↓*SNORA71B*, ↓*SPATS2*, ↓*ST7OT1*, ↓*TMEM217*, ↓*TOP2B*, ↑*TSPAN14*, ↓*TSPYL5*, ↓*UQCC*, ↑*WIPI1*	___________

^1^The biological processes categories were obtained from the GOA database. Genes (↑) up-regulated or (↓) down-regulated in MDS-RARS patients with respect to healthy individuals.

**Figure 2:**
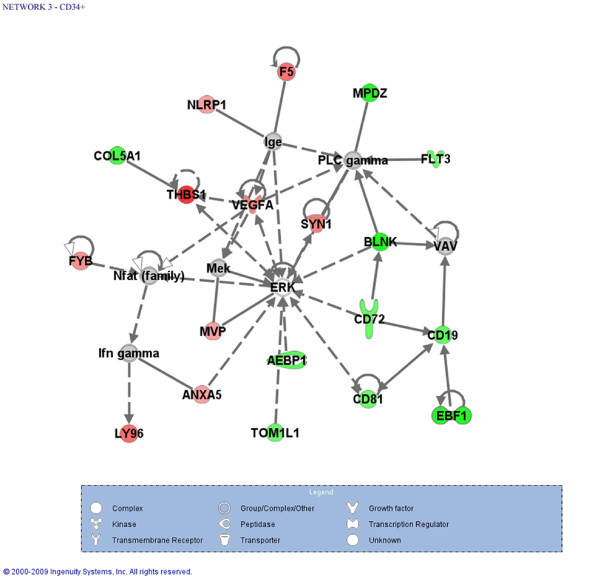
**Significantly enriched gene network of protein-coding genes with altered expression in CD34^+ ^cells**. Ingenuity Pathways Analysis identified that the top most significantly enriched (p < 0.001) functions of genes in this network were related to hematological system development and function, humoral immune response and tissue morphology. Gene color intensity indicates the degree of up-regulation (red) or of down-regulation (green) in CD34^+ ^cells of MDS-RARS patients in comparison to healthy individuals. Genes in gray were not identified as differentially expressed in our experiment and white genes were either not detected as expressed in these cells or not present in our oligoarray platform. Solid lines indicate direct interaction and dashed lines indirect interactions.

Ingenuity Pathways Analysis (IPA) was used for identifying enriched gene networks and functions among the differentially transcribed protein-coding genes. We identified 11 relevant networks that were significantly enriched (p-value < 0.001) with genes belonging to the MDS-RARS gene expression signature in CD34^+ ^cells (Additional file 2). Figure 2 shows a gene network involved in hematological system development and function, humoral immune response and tissue morphology.

**Figure 3:**
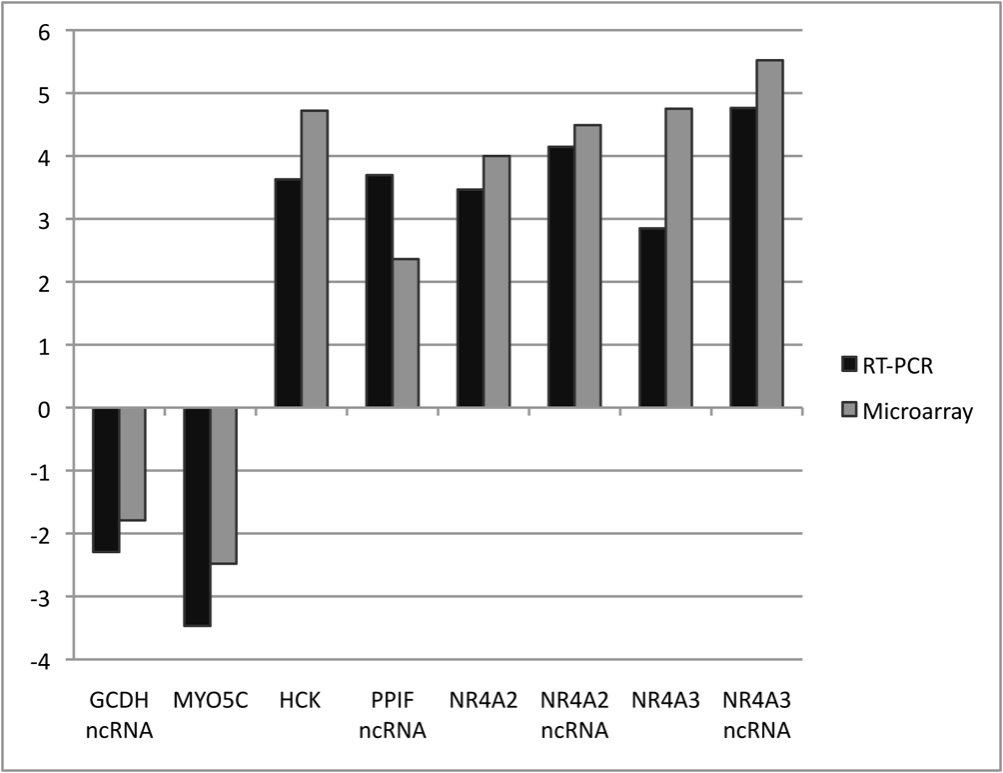
**Validation of gene expression data of CD34^+ ^cells by real-time RT-PCR**. Comparison of gene expression levels by real-time RT-PCR (black bars) and microarray experiments (gray bars) for eight select genes (names indicated at bottom) in CD34^+ ^cells of MDS-RARS patients and healthy individuals. Positive and negative fold change values indicate the up- or down-regulation of expression in MDS-RARS patients in relation to the expression in healthy individuals, respectively.

Eight transcripts were chosen to validate microarray data by real-time RT-PCR. RNA extracted from CD34^+ ^cells from 5 MDS-RARS patients (nos. 2-6; Table 1) was calculated as fold change compared with healthy controls. All transcripts were confirmed by real-time RT-PCR (Figure 3).

### Protein-coding and ncRNA transcript expression profiles in stromal cells

**Figure 4:**
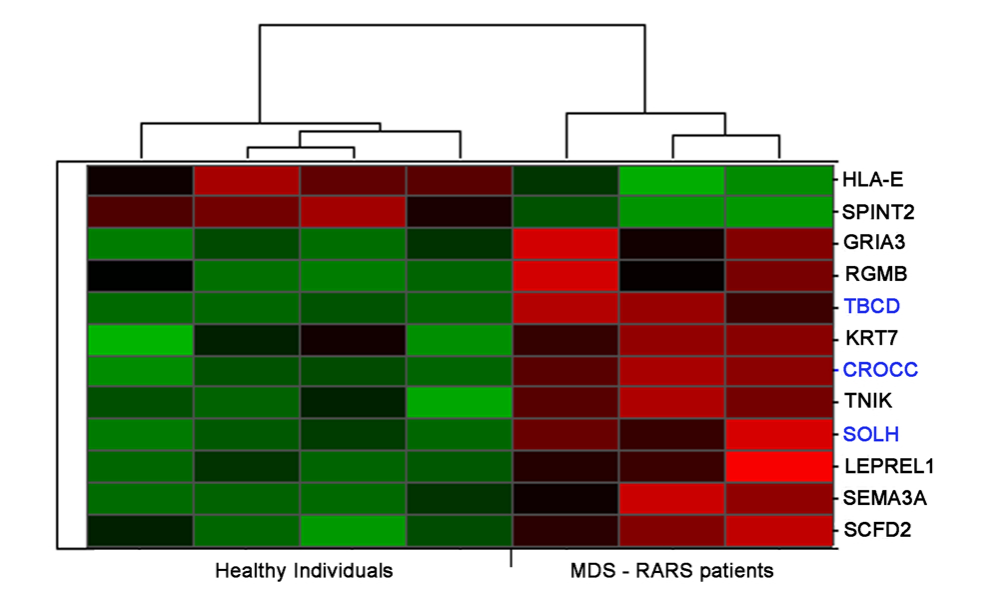
**Differentially expressed transcripts in stromal cells of MDS-RARS patients and healthy individuals**. The panel shows the expression matrix of 12 significantly differentially expressed genes in stromal cells of MDS-RARS patients compared to healthy individuals (SAM FDR <5% and fold change ≥1.7). Each row represents a single gene (9 protein-coding genes with names in black, and 3 ncRNAs in blue) and each column represents a separate stromal donor sample. Donor samples were clustered according to the correlation of expression profiles using the Unweighted Pair-Group Method, which resulted in two homogenous groups: MDS-RARS patients (3 columns at right) and healthy individuals (4 columns at left). Expression level of each gene is represented by the number of standard deviations above (red) or below (green) the average value for that gene across all samples. In MD-RARS patients, a total of 10 genes were up-regulated and 2 down-regulated.

Stromal cells obtained from three MDS-RARS patients (nos. 4-6; Table 1) were compared with stromal cells of healthy individuals using the same custom-designed combined intron-exon expression oligoarrays and significance analysis. SAM combined with patient leave-one-out cross validation identified 12 significantly (q-value ≤ 0.05) differentially expressed genes (10 up-regulated and 2 down-regulated in stromal cells of MDS-RARS patients) (Figure 4; Additional file 3), of which 3 were ncRNAs (up-regulated in MDS-RARS patients) (Table 5). The low number of differentially expressed genes was mostly due to the high homogeneity of stromal cells from patients and donors (correlation coefficient between all donor and patient stromal samples = 0.93, contrasted to 0.9 of CD34^+ ^cells, p = 10^-5^). The signature expression profile of protein-coding transcripts in MDS-RARS stromal cells revealed genes related to several biological processes, such as cell motility, DNA replication, protein amino acid phosphorylation and protein transport (Table 4).

**Table 5:** ncRNAs differentially expressed in stromal cells of MDS-RARS patients in comparison to healthy individuals.

Gene Locus Name^1^	Locus ID	ncRNA Probe Coordinates	Probe Strand	Type	Orientation in relation to protein coding gene	q value^2^	Fold Change
**Up-regulation in MDS-RARS**							
*SOLH*	6650	chr16:529804-529859	+	Intronic	Sense	0.000	4.40
*CROCC*	9696	chr1:16968607-16968658	+	Intergenic	Sense	0.038	2.40
*TBCD*	6904	chr17:78399392-78399451	+	Intronic	Sense	0.042	2.00

^1 ^Gene locus name for intronic ncRNA is that of the protein-coding gene of the same locus; intergenic ncRNA is annotated with the name of the nearest protein-coding gene in that chromosome.

^2 ^Minimum significance among all patient Leave-one-out analyses

**Figure 5:**
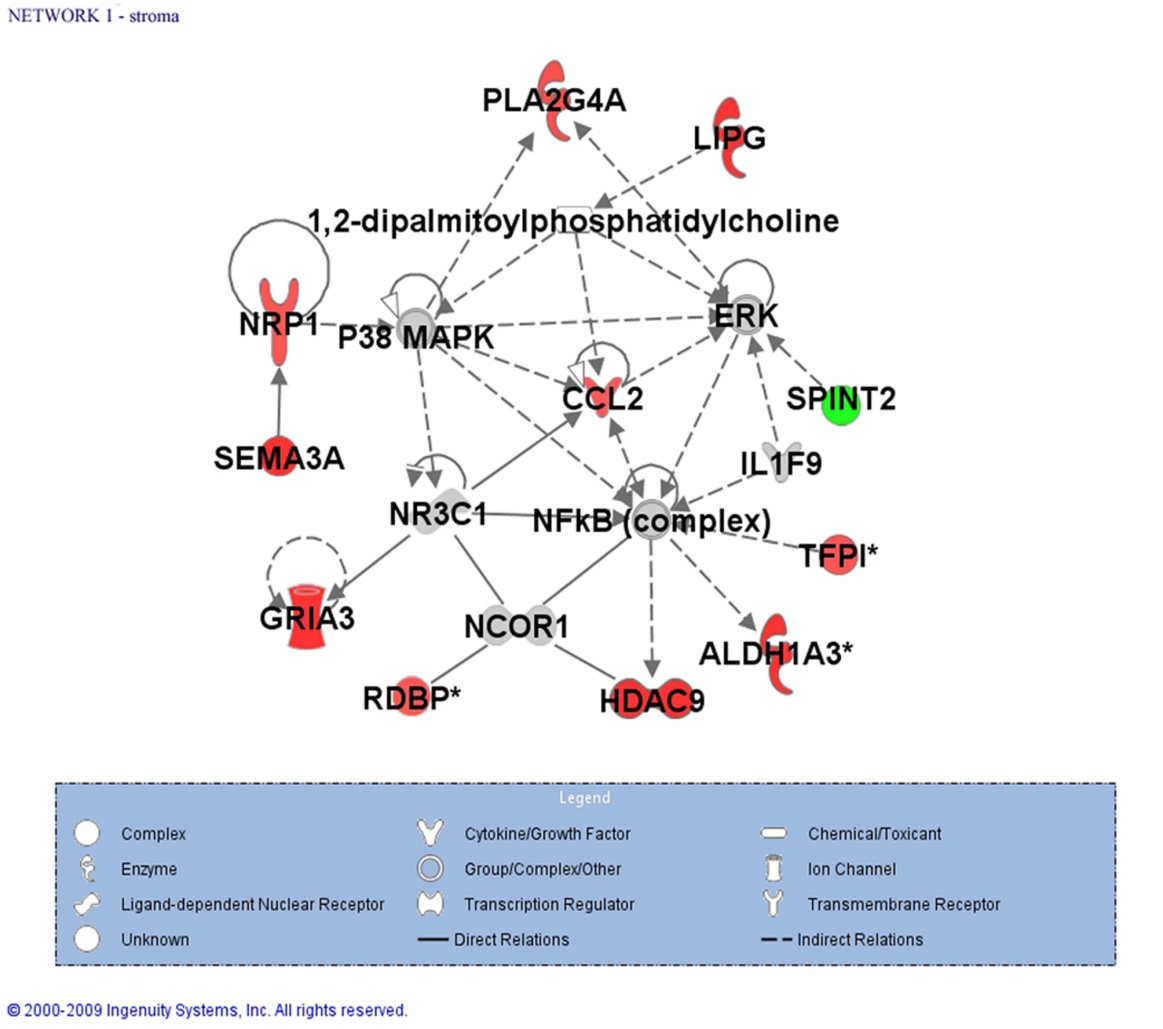
**Most enriched gene network of protein-coding genes with altered expression in stromal cells**. Ingenuity Pathways Analysis identified that the top most significantly enriched (p <0.001) functions of genes in this network were related to cell morphology, cellular compromise and neurological disease. Gene color intensity indicates the degree of up-regulation (red) or of down-regulation (green) in stromal cells of MDS-RARS patients in comparison to healthy individuals. Genes in gray were not identified as differentially expressed in our experiment and white genes were either not detected as expressed in these cells or not present in our oligoarray platform. Solid lines indicate direct interaction and dashed lines indirect interactions.

Ingenuity Pathways Analysis (IPA) of stromal cell genes statistically differentially expressed between MDS-RARS patients and healthy individuals (fold change ≥ 1.7; q-value ≤ 0.15 in all patient leave-one-out cross-validation analyses) identified two significantly enriched gene networks (p-value < 0.001) (Additional file 2). The most significantly enriched gene network involves genes related to cell morphology, cellular compromise, and neurological disease (Figure 5).

**Figure 6:**
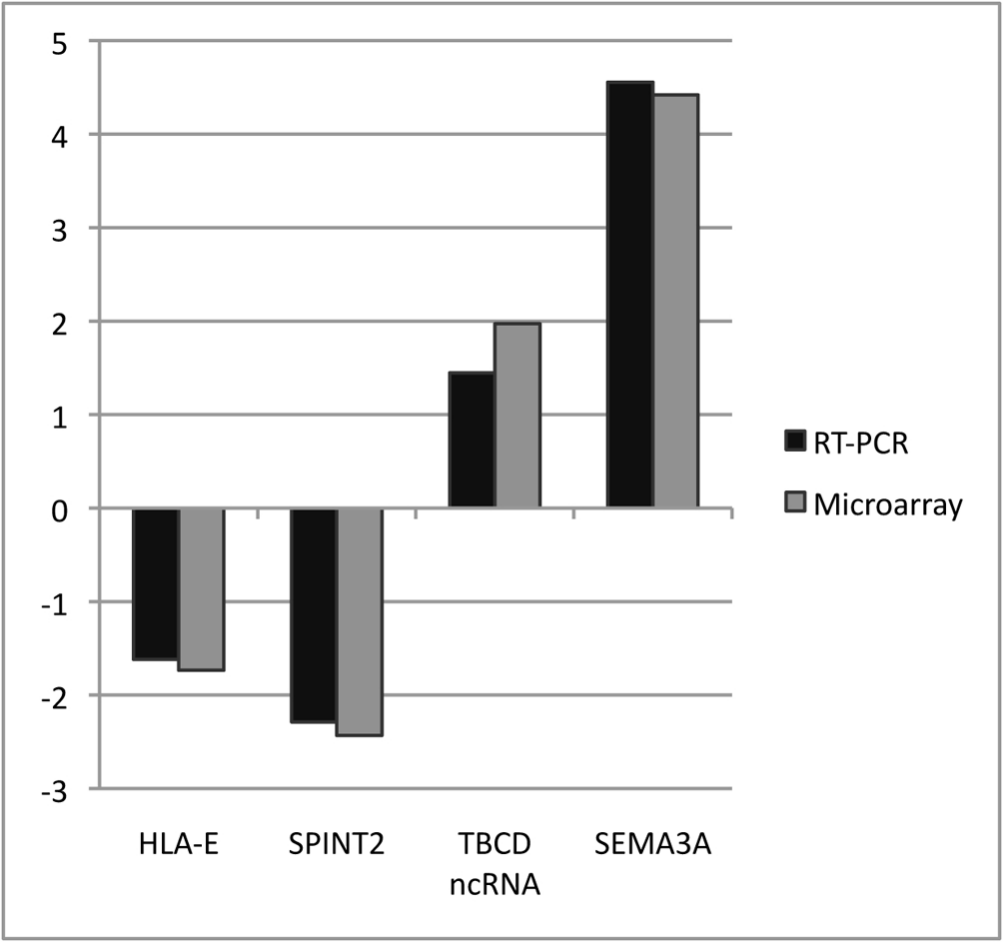
**Validation of gene expression data of stromal cells by real-time RT-PCR**. Comparison of gene expression obtained from real-time RT-PCR (black bars) and microarray experiments (gray bars) for four select genes (names indicated at bottom) in stromal cells of MDS-RARS patients and of healthy individuals. The positive and negative fold change values indicate up- or down-regulation of expression in MDS-RARS patients in relation to the expression in healthy individuals, respectively.

Four transcripts were chosen to validate microarray data by real-time RT-PCR. RNA extracted from stromal cells from 5 MDS-RARS patients (nos. 3-7; Table 1) was used for validation studies; all transcripts were confirmed by real-time RT-PCR (Figure 6).

## Discussion

In this study, we used the 44 k intron-exon oligoarray and stringent statistical criteria to determine the protein-coding and intronic non-coding transcript expression profiles in CD34^+ ^and stromal cells of MDS-RARS patients and healthy individuals. We herein validated the expression of a set of selected transcripts by real time RT-PCR in five MDS-RARS patients, however future confirmation in a larger group of MDS-RARS cases is warranted. Pathway analyses of differential protein-coding transcripts pointed to new genetic networks that are altered in both CD34^+ ^and stromal cells of MDS-RARS patients (Additional file 2).

MDS are characterized by hematopoietic insufficiency associated with cytopenia, leading to severe morbidity in addition to increased risk of leukemia transformation [46]. The exact stage of CD34^+ ^progenitor cells involved in the process of MDS and transformation to AML are still in debate. Bone marrow microenvironment contributes to regulate self-renewal, commitment, differentiation, proliferation and the dynamics of apoptosis of hematopoietic progenitors [47], and CD34^+ ^progenitor cells are known to be severely impacted in MDS by the composition of micro environmental stimuli [48]. Detection of differentially expressed transcripts in MDS-RARS stromal cells suggests that these transcripts could contribute to maintain CD34^+ ^cells.

Pathways analysis of the set of protein-coding transcripts altered in CD34^+ ^cells of MDS-RARS patients revealed an important gene network related to hematological disease, including *BLNK, CD19, CD72, CD81*, *EBF1, F5, FLT3, LY96, MPDZ *and *THBS1*. Down-regulation in MDS-RARS of genes related to the B cell receptor signaling pathway were found in our results (*BLNK, CD19 *and *CD72*), and the low expression of several genes involved in B lymphocyte development, corroborate the hypothesis that early MDS can be defined by a B-cell progenitor defect [49].

The abnormal expression of genes encoding mitochondrial proteins involved in iron metabolism has been characterized in MDS-RARS [7,50]. The present study found 3 different ncRNA transcripts with altered expression in the CD34^+ ^cells of MDS-RARS patients, from gene loci of mitochondrial proteins (*AASS, GCDH *and *PPIF*) and 6 modulated mitochondrial protein-coding transcripts. In addition, corroborating our findings, the down regulation of iron transporter *ABCB7 *has been described in CD34^+ ^cells of patients MDS-RARS, indicating that low *ABCB7 *levels would contribute to abnormal mitochondrial iron homeostasis [43]. Furthermore, genes related to heme biosynthesis pathway and transferrin trafficking (*PXDN *and *MYO5C*, respectively), were down regulated in MDS-RARS CD34^+ ^cells. These differentially expressed genes could be related to the iron accumulation observed in mitochondria of RARS patients.

One of the mechanisms that contribute to hypercellular marrow and peripheral blood cytopenia of patients with early stage MDS is the significant increase in apoptosis of hematopoietic cells [51]. The higher expression of Fas-FasL system found in MDS plays a role in inducing MDS bone marrow apoptosis and works in both an autocrine (hematopoietic cell-hematopoietic cell interaction) or paracrine (hematopoietic cell-stromal cell interaction) pattern [52]. The protein encoded by *SEMA3A *(Class 3 semaphorins), a secreted member of the semaphorin family involved in axonal guidance, organogenesis, angiogenesis, and highly expressed in several tumor cells [53,54], has recently been demonstrated to be an important determinant of leukemic cells sensitivity to Fas-mediated apoptosis signal [55]. Furthermore, Sema3A has already been described to act through different signaling pathways to control neural progenitor cell repulsion activating Erk1/2 or apoptosis process involving p38MAPK [56]. Surprisingly, *SEMA3A *is present in both affected networks of MDS-RARS stromal cells (see Additional file 2), suggesting participation of this gene in diverse abnormalities implicated in the modification of hematopoietic cells development and apoptosis in MDS [12,13].

The non-coding expression profiles of CD34^+ ^and stromal cells of MDS-RARS were clearly distinct from those obtained from CD34^+ ^and stromal cells of healthy controls, representing 30% and 25% of the total amount of differentially expressed genes in CD34^+ ^and stromal cells of MDS-RARS patients, respectively. Currently, evidence of the biological roles played by ncRNA have increased, especially those transcribed from partially conserved introns of protein-coding genes [57]. Recently, eosinophil granule ontogeny (EGO) has been shown to involve an ncRNA expressed during IL-5 stimulation, whose function is to regulate MBP granule protein and *EDN *mRNA levels [58].

Interestingly, our results showed 13 differentially expressed ncRNA transcripts in CD34^+ ^cells of MDS-RARS patients for which there was a simultaneous change in expression of the protein-coding gene in the corresponding locus: for 7 of them both the ncRNA and the protein-coding gene were simultaneously down-regulated in MDS-RARS, 5 were up-regulated, and in one gene locus the TIN ncRNA was up-regulated whereas the protein-coding gene was down-regulated. Expression of both, protein-coding and non-coding pairs in the same locus, suggest that these intronic ncRNAs may act upon cis-regulatory factors, modulating the stability and/or processing of the corresponding protein-coding transcript, or even directly affecting the levels and/or the splicing of protein-coding isoforms [27,59]. We found 2 altered genes of the nuclear receptor subfamily 4, group A (*NR4A2 *e *NR4A3*), known to be involved in T-cell apoptosis, brain development, and vascular disease [60], and both showed a simultaneous up-regulation of the protein-coding and the ncRNA from the same locus in MDS-RARS, suggesting that these ncRNAs could be involved in the control of protein coding expression of this gene family in MDS-RARS patients.

## Conclusion

The presence of intronic ncRNA transcripts differentially expressed in CD34^+ ^and stromal cells may shed light upon the not yet fully understood molecular mechanisms involved in the heterogeneity of myelodysplastic syndromes and suggest that ncRNAs may play a role during disease development. Characterization of those ncRNA transcripts would contribute to a better understanding of MDS-RARS, or even towards the development of biomarkers and therapeutic targets.

## Competing interests

The authors declare that they have no competing interests.

## Authors' contributions

MBO was the principal investigator and takes the primary responsibility for the paper. YMB performed the microarray analysis for this study. FT recruited the patients. FFC, SVA and STOS coordinated the research. MBO, YMB, SVA and STOS wrote the paper. All authors have read and approved the final manuscript.

## Pre-publication history

The pre-publication history for this paper can be accessed here:

http://www.biomedcentral.com/1755-8794/3/30/prepub

## Supplementary Material

Additional file 1 **Transcripts with altered expression in CD34^+ ^cells of MDS-RARS**. This file can be viewed with: Adobe Acrobat Reader Click here for file

Additional file 2 **The genetic networks of protein-coding transcripts differently expressed of MDS-RARS patients in relation to healthy individuals, obtained from IPA analysis**. This file can be viewed with: Adobe Acrobat Reader Click here for file

Additional file 3 **Transcripts with altered expression in stromal cells of MDS-RARS** . This file can be viewed with: Adobe Acrobat Reader Click here for file
