# Supplementary material for: Identification of protein-coding and non-coding RNA expression profiles in CD34+ and in stromal cells in refractory anemia with ringed sideroblasts
Source: BMC Med Genomics. 2010 Jul 15;3:30. doi: 10.1186/1755-8794-3-30 (PMC2914047; doi:10.1186/1755-8794-3-30)
Supplement: Additional file 1 — Transcripts with altered expression in CD34+ cells of MDS-RARS. This file can be viewed with: Adobe Acrobat Reader [file 1755-8794-3-30-S1.PDF]

Additional file 1. Transcripts with altered expression in CD34+ cells of MDS-RARS.

Gene Locus name [1]	Locus ID	Probe Coordinate	Probe Strand	Type	Orientation in relation to the protein coding gene	q value[2]	Fold Change
**down-regulated in MDS-RARS**							
AADAT	51166	chr4:171356547-171356606	-	Exonic		0.001	-2.38
AASS	10157	chr7:121310573-121310613	-	Exonic		0.000	-2.99
AASS	10157	chr7:121332812-121332871	+	Intronic	Antisense	0.001	-1.79
ABCB7	22	chrX:74056355-74056414	-	Exonic		0.000	-1.75
ACSM3	6296	chr16:20700539-20700598	+	Exonic		0.000	-2.07
ACSM3		chr16:20715193-20715252	+	Exonic		0.000	-2.00
AEBP1	165	chr7:43927062-43927121	+	Exonic		0.000	-2.05
AFF1	4299	chr4:88268668-88268724	+	Intronic	Sense	0.000	-1.83
AFF3	3899	chr2:99640471-99640526	-	Exonic		0.001	-2.48
AFF3	3899	chr2:99698165-99698216	-	Intronic	Sense	0.004	-1.76
AHI1	54806	chr6:135791459-135791499	-	Exonic		0.000	-2.07
ALS2CR4	65062	chr2:202310578-202310637	-	Exonic		0.001	-1.81
ANK3	288	chr10:61459558-61459591	-	Exonic		0.000	-3.01
ASAP2	8853	chr2:9299234-9299293	+	Intronic	Sense	0.003	-2.14
ASXL1	171023	chr20:30410722-30410781	+	Intronic	Sense	0.001	-1.94
AUTS2	26053	chr7:69200166-69200225	+	Exonic		0.000	-3.33
AUTS2	26053	chr7:69702065-69702124	+	Exonic		0.001	-2.67
BACH2	60468	chr6:90693326-90693385	-	Exonic		0.001	-3.28
BLNK	29760	chr10:97961400-97961459	-	Intronic	Sense	0.000	-3.59
BLNK	29760	chr10:97946676-97946735	-	Exonic		0.000	-2.88
C13orf18	80183	chr13:45816907-45816966	-	Exonic		0.003	-2.06
C16orf67	79014	chr16:31625733-31625792	+	Exonic		0.000	-2.83
C16orf67	79014	chr16:31624199-31624258	+	Exonic		0.001	-1.91
C1orf21	81563	chr1:181329653-181329712	+	Exonic		0.001	-2.62
C5	727	chr9:120794621-120794680	-	Exonic		0.001	-1.86
C5orf13	9315	chr5:111094583-111094642	+	Intronic	Antisense	0.000	-1.86
C9orf58	83543	chr9:131027887-131027946	+	Exonic		0.001	-1.92
CCDC136	64753	chr7:128049619-128049675	+	Exonic		0.003	-4.12
CCDC136	64753	chr7:128048785-128048844	+	Exonic		0.004	-2.10
CD19	930	chr16:28858101-28858160	+	Exonic		0.002	-1.92
CD81	975	chr11:2374760-2374819	+	Exonic		0.000	-1.77
CEP290	80184	chr12:87007495-87007554	-	Exonic		0.001	-1.81
CLIP3	25999	chr19:41197577-41197636	-	Exonic		0.003	-2.00
COL5A1	1289	chr9:134960497-134960556	+	Exonic		0.000	-2.24
CPSF6	11052	chr12:67954327-67954386	-	Intronic	Antisense	0.000	-2.16
CPSF6	11052	chr12:67954327-67954386	-	Intronic	Antisense	0.000	-1.97
CYFIP2	26999	chr5:156754713-156754772	+	Exonic		0.000	-1.78
CYYR1	116159	chr21:26760551-26760610	-	Exonic		0.002	-2.01
DLG3	1741	chrX:69508275-69508334	+	Exonic		0.000	-2.63
DPY19L2	283417	chr12:62260709-62260753	-	Exonic		0.000	-1.86
DST	667	chr6:56456393-56456452	-	Exonic		0.001	-2.42
E2F7	144455	chr12:75917988-75918047	-	Exonic		0.000	-2.19
EBF1	1879	chr5:158058593-158058652	-	Exonic		0.003	-3.53
ECHDC2	55268	chr1:53073926-53073985	-	Exonic		0.001	-1.80

ELP2	55250	chr18:32008518-32008577	+	Exonic		0.001	-1.76
ERAP1	51752	chr5:96279013-96279072	+	Exonic		0.004	-9.47
ERO1LB	56605	chr1:232705155-232705214	-	Intronic	Antisense	0.000	-1.98
FAAH	2166	chr1:46588511-46588546	+	Exonic		0.000	-2.40
FLT3	2322	chr13:27475993-27476051	-	Exonic		0.000	-1.82
GABPB1	2553	chr15:48447272-48447331	+	Exonic		0.000	-2.55
GABPB1	2553	chr15:48448783-48448842	+	Intergenic	Antisense	0.003	-1.85
GABPB1	2553	chr16:11871200-11871259	-	Intergenic	Antisense	0.003	-1.79
GCDH	2639	chr19:12871038-12871097	-	Intronic	Antisense	0.000	-1.79
HHAT	55733	chr1:207237736-207237795	+	Exonic		0.000	-2.29
HS3ST1	9957	chr4:11076939-11076998	-	Exonic		0.000	-2.09
IGSF10	285313	chr3:152637104-152637163	+	Intergenic	Sense	0.000	-2.19
JAM2	58494	chr21:26000228-26000269	+	Exonic		0.000	-3.14
KCNE1L	23630	chrX:108673158-108673217	-	Exonic		0.000	-2.36
KCNMB3	27094	chr3:180451233-180451268	-	Exonic		0.000	-2.70
KCNMB4	27345	chr12:69110890-69110949	+	Exonic		0.001	-2.33
KLHL5	51088	chr4:38941503-38941562	+	Exonic		0.004	-2.92
LAMB2	3913	chr3:49133567-49133626	-	Exonic		0.004	-1.93
LAMC1	3915	chr1:179846063-179846122	+	Exonic		0.003	-1.82
LAYN	143903	chr11:110936670-110936729	+	Exonic		0.001	-2.24
LEF1	51176	chr4:109454925-109454984	+	Intergenic	Antisense	0.001	-2.80
LOC400713	400713	chr19:57579855-57579914	+	Exonic		0.003	-1.84
LOC441242	441242	chr7:63977853-63977912	-	Intergenic	Sense	0.003	-1.82
LRIG1	26018	chr3:66512287-66512346	-	Exonic		0.003	-1.81
MED12L	116931	chr3:152412757-152412816	-	Exonic		0.000	-2.45
MGC29506	51237	chr5:138751442-138751501	-	Exonic		0.003	-1.77
MMP11	4320	chr22:22450827-22450886	+	Exonic		0.001	-1.78
MORN1	79906	chr1:2326963-2327014	-	Intronic	Sense	0.003	-2.15
MPDZ	8777	chr9:13095883-13095942	-	Exonic		0.001	-3.15
MYO1D	4642	chr17:27843945-27844004	-	Exonic		0.001	-2.27
MYO5C	55930	chr15:50271936-50271995	-	Exonic		0.000	-2.48
NASP	4678	chr1:45744001-45744060	+	Intronic	Sense	0.001	-2.10
NASP	4678	chr1:45749924-45750086	+	Exonic		0.001	-1.93
NASP	4678	chr1:45741247-45741306	+	Exonic		0.002	-1.73
NAV1	89796	chr1:198504378-198504437	+	Exonic		0.002	-1.74
NEIL1	79661	chr15:73434282-73434341	+	Intronic	Sense	0.000	-4.45
NEIL1	79661	chr15:73434051-73434110	+	Exonic		0.000	-3.72
NEIL1	79661	chr15:73434426-73434485	+	Exonic		0.001	-3.36
NKD2	85409	chr5:1091719-1091778	+	Exonic		0.004	-2.55
NOL7	51406	chr6:13722339-13722397	-	Exonic		0.000	-1.75
NPY	4852	chr7:24104524-24104583	+	Exonic		0.000	-3.88
NPY	4852	chr7:24102358-24102417	+	Exonic		0.000	-3.08
NSMCE4A	54780	chr10:123717158-123717201	-	Exonic		0.001	-1.74
NUP153	9972	chr6:17815444-17815503	+	Intergenic	Sense	0.001	-2.04
PAG1	55824	chr8:82068816-82068875	-	Intronic	Sense	0.001	-1.83
PCDH9	5101	chr13:66698353-66698412	-	Exonic		0.004	-2.33
PDZD2	23037	chr5:32146500-32146559	+	Exonic		0.000	-1.81
PIAS2	9063	chr18:42643244-42643303	-	Intergenic	Sense	0.000	-2.05
PLS3	5358	chrX:114707296-114707355	+	Exonic		0.000	-2.22
POU2AF1	5450	chr11:110728214-110728273	-	Exonic		0.002	-2.31
PRG4	10216	chr1:183013558-183013617	-	Intronic	Antisense	0.000	-1.86
PSD3	23362	chr8:18429333-18429388	-	Exonic		0.003	-2.06

PTGR1	22949	chr9:111420724-111420783	-	Exonic		0.003	-1.81
PXDN	7837	chr2:1607456-1607515	-	Exonic		0.000	-4.14
PXDN	7837	chr2:1606432-1606473	-	Exonic		0.001	-4.14
RBM4B	83759	chr11:66192338-66192397	+	Intronic	Sense	0.001	-1.73
RIMS3	9783	chr1:40755662-40755721	-	Exonic		0.000	-2.17
ROBO1	6091	chr3:78729485-78729544	-	Exonic		0.000	-4.86
SEC63	11231	chr6:108329351-108329410	-	Exonic		0.000	-1.77
SH2D4B	387694	chr10:82393858-82393917	+	Exonic		0.001	-2.86
SLC12A2	6558	chr5:127550303-127550362	+	Exonic		0.000	-1.94
SLC39A8	64116	chr4:103540129-103540188	-	Exonic		0.000	-1.73
SNORA71B	26776	chr20:36482692-36482751	-	Exonic		0.001	-1.78
SNORA71B	26776	chr20:36482692-36482751	-	Intergenic	Sense	0.001	-1.77
SOCS2	8835	chr12:92472267-92472326	+	Exonic		0.000	-1.75
SPATS2	65244	chr12:48204855-48204914	+	Exonic		0.000	-1.74
ST7OT1	93653	chr7:116186492-116186551	-	Intergenic	Sense	0.004	-2.13
TLE1	7088	chr9:81478431-81478490	-	Intronic	Sense	0.000	-2.30
TLE1	7088	chrX:64411696-64411755	+	Intronic	Sense	0.000	-2.12
TLE1	7088	chr9:81478491-81478550	-	Intronic	Sense	0.000	-2.10
TLE1	7088	chr9:81428672-81428731	-	Exonic		0.000	-1.87
TMEM217	221468	chr6:37288040-37288099	-	Exonic		0.000	-1.84
TNFAIP3	7128	chr6:138230463-138230522	-	Intronic	Antisense	0.000	-2.21
TNFRSF21	27242	chr6:47308509-47308568	-	Exonic		0.001	-2.10
TOM1L1	10040	chr17:50393842-50393901	+	Exonic		0.001	-1.81
TOP2B	7155	chr3:25661877-25661936	-	Exonic		0.003	-2.01
TOP2B	7155	chr3:25614718-25614777	-	Exonic		0.000	-1.85
TOP2B	7155	chr3:25615965-25616024	-	Exonic		0.000	-1.81
TSPYL5	85453	chr8:98354961-98355020	-	Exonic		0.000	-2.00
UHRF1	29128	chr19:4913065-4913124	+	Exonic		0.000	-1.76
UNC13B	10497	chr9:35394491-35394550	+	Exonic		0.001	-1.81
UQCC	55245	chr20:33353970-33354029	-	Exonic		0.002	-1.84
ZNF367	195828	chr9:96227967-96228026	-	Exonic		0.000	-3.00
ZNF711	7552	chrX:84333012-84333071	+	Exonic		0.000	-2.33
ZNF76	7629	chr6:35364312-35364371	+	Intronic	Sense	0.002	-2.02
ZNF91	7644	chr19:23334238-23334297	-	Exonic		0.000	-2.02
**up-regulated in MDS-RARS**							
AADACL1	57552	chr3:173831278-173831337	-	Exonic		0.002	1.96
ACPP	55	chr3:133551496-133551544	+	Exonic		0.004	2.46
ADCY7	113	chr16:48907161-48907220	+	Exonic		0.001	1.92
AKAP13	11214	chr15:84021272-84021331	+	Exonic		0.003	2.33
ANK3	288	chr10:61625808-61625867	-	Intronic	Sense	0.004	2.30
AOAH	313	chr7:36343264-36343309	-	Exonic		0.004	1.97
ARHGEF10L	55160	chr1:17769433-17769492	+	Exonic		0.002	5.07
B3GNT5	84002	chr3:184455731-184455790	+	Intronic	Sense	0.001	2.21
CALML4	91860	chr15:66273414-66273473	-	Exonic		0.005	1.72
CC2D1A	54862	chr19:13891701-13891760	-	Intronic	Antisense	0.002	1.88
CCDC146	57639	chr7:76467266-76467325	-	Intronic	Antisense	0.003	4.03
CD44	960	chr11:35184235-35184294	-	Intronic	Antisense	0.000	1.83
COTL1	23406	chr16:83156791-83156850	-	Exonic		0.004	1.89
CTSH	1512	chr15:77001264-77001323	-	Exonic		0.002	2.91
CTSH	1512	chr15:77007119-77007178	-	Exonic		0.000	2.79
CTSS	1520	chr1:147535553-147535603	-	Exonic		0.000	3.07
CTSS	1520	chr1:147540640-147540699	-	Exonic		0.000	2.68

CYB5R1	51706	chr1:199642932-199642991	-	Exonic		0.001	2.34
CYB5R1	51706	chr1:199641923-199641982	-	Exonic		0.003	1.75
CYBASC3	220002	chr11:60883741-60883792	-	Intronic	Sense	0.002	2.21
DAGLBETA	221955	chr7:6274325-6274384	+	Intronic	Antisense	0.003	1.78
DDX3X	1654	chrX:40963266-40963325	+	Exonic		0.002	2.77
DDX3X	1654	chrX:40963207-40963266	+	Exonic		0.003	1.86
DDX3Y	8653	chrY:13465993-13466052	+	Exonic		0.003	4.26
DHX38	9785	chr16:70696571-70696630	+	Intronic	Sense	0.000	2.11
EHBP1L1	254102	chr11:65104384-65104443	-	Intronic	Antisense	0.001	2.38
EHBP1L1	254102	chr11:65114552-65114611	+	Exonic		0.000	1.91
ETV3	2117	chr1:153916337-153916396	-	Exonic		0.002	1.78
FAM50A	9130	chrX:153324988-153325047	+	Intronic	Antisense	0.002	2.05
FGD6	55785	chr12:93977621-93977680	-	Exonic		0.002	1.75
FGFR1OP2	26127	chr12:27004715-27004774	+	Exonic		0.000	1.97
FN1	2335	chr2:216086310-216086369	+	Intronic	Antisense	0.000	2.71
FRAS1	80144	chr4:79383367-79383426	+	Intronic	Sense	0.004	1.85
HCK	3055	chr20:30140061-30140108	+	Exonic		0.001	4.72
HVCN1	84329	chr12:109549467-109549526	-	Exonic		0.003	1.83
ICA1	3382	chr7:7932382-7932433	-	Intronic	Sense	0.000	1.86
IFI30	10437	chr19:18147121-18147180	+	Exonic		0.001	5.43
IFI30	10437	chr19:18149840-18149899	+	Exonic		0.000	4.35
IFI30	10437	chr19:18147121-18147180	-	Intronic	Antisense	0.004	2.12
IL10RA	3587	chr11:117375219-117375278	+	Exonic		0.005	2.61
ITGAX	3687	chr16:31301452-31301511	+	Exonic		0.002	3.46
JAZF1	221895	chr7:27643617-27643676	-	Exonic		0.001	1.95
JHDM1D	80853	chr7:139238230-139238289	-	Exonic		0.000	2.09
LFNG	3955	chr7:2341212-2341271	+	Exonic		0.000	3.76
LRP1	4035	chr12:55892213-55892272	+	Exonic		0.005	4.03
LY96	23643	chr8:75103765-75103824	+	Exonic		0.001	2.68
MARCH1.	55016	chr4:164805948-164806007	-	Intergenic	Sense	0.002	3.77
NCOA3	8202	chr20:45714557-45714616	-	Intronic	Antisense	0.003	1.86
NCOA4	8031	chr10:51259279-51259338	+	Exonic		0.003	2.03
NR4A2	4929	chr2:157011025-157011084	-	Intronic	Sense	0.002	4.49
NR4A2	4929	chr2:157008881-157008940	-	Exonic		0.002	3.51
NR4A2	4929	chr2:157012210-157012269	+	Intronic	Antisense	0.004	2.15
NR4A3	8013	chr9:99668743-99668802	+	Intronic	Sense	0.001	5.52
NR4A3	8013	chr9:99675306-99675365	+	Exonic		0.002	5.05
NR4A3	8013	chr9:99668564-99668623	+	Exonic		0.000	4.45
OAS1	4938	chr12:111817181-111817240	+	Exonic		0.002	3.80
PHLDA2	7262	chr11:2906272-2906331	-	Exonic		0.002	2.61
PKM2	5315	chr15:70288031-70288090	+	Intronic	Antisense	0.000	1.81
PPIF	10105	chr10:80781110-80781169	-	Intronic	Antisense	0.001	2.36
PPIF	10105	chr10:80781110-80781169	+	Intronic	Sense	0.003	2.29
PPP1R15A	23645	chr19:54068439-54068498	-	Intronic	Antisense	0.002	1.82
RAD51L1	5890	chr14:67951712-67951763	-	Intronic	Antisense	0.001	1.77
RALGPS1	9649	chr9:126945696-126945747	+	Intronic	Antisense	0.000	2.70
RP5-1022P6.2	56261	chr20:5491968-5492027	-	Intronic	Sense	0.002	1.81
RXRA	6256	chr9:134554548-134554607	+	Exonic		0.000	4.04
S100A11	6282	chr1:148818292-148818351	-	Exonic		0.003	2.91
S100A4	6275	chr1:150330268-150330327	+	Intronic	Antisense	0.001	1.71
SERPINA1	5265	chr14:93919199-93919258	-	Exonic		0.001	5.47
SERPINA1	5265	chr14:93914544-93914603	-	Exonic		0.001	4.55

SERPINA1	5265	chr14:93919199-93919258	+	Intronic	Antisense	0.001	2.51
SESTD1	91404	chr2:179802621-179802672	+	Intronic	Antisense	0.000	1.91
SGK1	6446	chr6:134532349-134532408	-	Exonic		0.001	2.96
SGSH	6448	chr17:75797932-75797991	-	Exonic		0.001	3.04
SLC11A2	4891	chr12:49679406-49679465	-	Exonic		0.000	2.09
SLC37A2	219855	chr11:124464243-124464302	+	Exonic		0.001	4.06
SLC43A2	124935	chr17:1440846-1440886	-	Exonic		0.000	3.02
SLC8A1	6546	chr2:40251076-40251135	-	Exonic		0.003	2.01
SNX20	124460	chr16:49264159-49264218	-	Intronic	Sense	0.000	1.88
THBS1	7057	chr15:37668391-37668450	+	Intronic	Sense	0.001	2.77
TMBIM6	7009	chr12:48437217-48437268	+	Intronic	Sense	0.001	1.85
TNFRSF1B	7133	chr1:12202541-12202600	+	Exonic		0.000	4.31
TSPAN14	81619	chr10:82267911-82267970	+	Exonic		0.000	1.97
VCAN	1462	chr5:82912058-82912117	+	Exonic		0.000	5.26
VEGFA	7422	chr6:43854201-43854612	+	Exonic		0.003	2.36
WIPI1	55062	chr17:63936613-63936672	-	Exonic		0.000	2.38
WIPI1	55062	chr17:63929049-63929108	-	Exonic		0.004	2.17
ZSCAN10	84891	chr16:3082319-3082378	-	Intronic	Sense	0.004	2.24

[1] Gene locus name for intronic ncRNA is that of the protein-coding gene of the same locus; intergenic ncRNA is annotated with the name of the nearest protein-coding gene in that chromosome

[2]Minimum significance among all patient Leave-one-out analyses.
