# Supplementary material for: Identification of protein-coding and non-coding RNA expression profiles in CD34+ and in stromal cells in refractory anemia with ringed sideroblasts
Source: BMC Med Genomics. 2010 Jul 15;3:30. doi: 10.1186/1755-8794-3-30 (PMC2914047; doi:10.1186/1755-8794-3-30)
Supplement: Additional file 2 — The genetic networks of protein-coding transcripts differently expressed of MDS-RARS patients in relation to healthy individuals, obtained from IPA analysis. This file can be viewed with: Adobe Acrobat Reader [file 1755-8794-3-30-S2.PDF]

Additional file 2. The genetic networks of protein-coding transcripts differently expressed of MDS-RARS patients in relation to healthy individuals, obtained from IPA analysis.

Network	Molecules in Network	Top Functions	-log10 (p value)	Focus Molecules
**In CD34[+] cells of MDS-RARS (FDR <5% and fold≥1.7)**				
1	↓BACH2, Ck2, ↑CREB5, ↑CYBB, ↑DUSP1, E2F7, JINK1/2, ↓JUN, ↓LEF1, ↓MMP11, NADPH oxidase, ↓NASP, ↑NCOA4, NFkB (complex), ↓NFKBIZ, Notch, ↑NR4A2, ↑PPIF, Proteasome, ↓PXDN, Rxr, ↑RXRA, ↑S100A11, ↑SAT1, ↑SERPINA1, ↑SLC11A2, ↑SMARCD3, Thyroid hormone receptor, ↓TLE1, Top2, ↓TOP2B, tyrosine kinase, ↓UHRF1, ↑VDR	Dermatological Diseases and Conditions, Genetic Disorder, Immunological Disease	44	24
2	Akt, ALP, ↓ANK3, ↑ASAH1, ↓AUTS2, Caspase, ↑CTSS, Cytochrome c, ↑DDX3X, ↓ELP2, Fgf, ↓GRK4, Hsp70, Hsp90, IFN Beta, Ikb, IKK, IL1, IL10RA, Interferon alpha, ↑ITGAX, LDL, ↑NR4A3, ↑OAS1, ↑OSBPL6, ↑SGK1, ↑SLC2A3, ↑SNCA, ↑SOCS2, ↑STAT, ↑STAT1, STAT5a/b, ↑TNFSF10, ↑TRAF1, Ubiquitin	Lipid Metabolism, Molecular Transport, Small Molecule Biochemistry	32	19
3	14-3-3, ↓AEBP1, ↑ANXA5, ↓BLNK, ↓CD19, ↓CD72, ↓CD81, ↓COL5A1, ↓EBF1, ERK, ↓F5, ↓FLT3, ↑FYB, Ifn gamma, Ige, Integrin, ↑LY96, MAP2K1/2, Mek, ↓MPDZ, ↑MVP, Nfat, ↑NLRP1, Pak, Pdgf, PLC gamma, Rac, Ras, Sphk, ↑SYN1, TCR, ↑THBS1, ↓TOM1L1, VAV, ↑VEGFA	Hematological System Development and Function, Humoral Immune Response, Tissue Morphology	29	19
4	↑AADACL1, ↓AFF3, amino acids, ↑ARHGEF10L, beta-estradiol, CDH11, ↓CLIP3, CTSL1, ↑CYB5R1, CYCS, CYP17A1, ↓DCLK2, ↓DLG3, ↑FGD6, glutamine, GRIN2C, HDL, HUNK, IRS1, KCNMA1, ↓KCNMB3, ↓KCNMB4, MIRN295, MIRN292, MPO, ↓MPPED2, ↑MYOF, NR4A1, ↑NR4A3, PPP3R1, RARS, ↑RXRA, ↓STXBP1, ↑TSHZ3, ↓ZNF91	Cardiovascular Disease, Cell Cycle, Auditory and Vestibular System Development and Function	27	17
5	ABL1, ↑AP1S2, ATF4, BRPF3, ↓CBX2, ↑CPEB4, CREB1, ↓EMCN, ↑FAM110A, ↓GCDH, GPR45, GRB2, ↓GTF2IRD1, ↓HLF, ↓HS3ST1, ↑JAZF1, MIRN202, MIRN24-1, NAP5, NEK8, NFS1, NR5A1, NRG1, ↓PALLD, ↓PLS3, ↑RCBTB2, retinoic acid, ↓RICH2, SCARF2, ↓SEC63, SELL, ↑SGSH, SNX8, TSKS, VPS13A	Gene Expression, Tissue Development, Cardiovascular Disease	25	16
6	ABR, ↑ATXN1, ↓BTG3, C12ORF23, ↓CCDC136, COIL, CPNE3, ↓DIDO1, DST, DVL3, F2, Itga4-Itgb1, ITGB1, ↓JAM2, ↓KIAA1841, ↓LRIG1, MIRN124, MIRN349, MIRN124-1, MIRN134, MIRN212, MIRN298, MYST3, ↓PLEKHG4, ↓PROSAPIP1, RAC1, RBMS1, ↓RIMS3, ↑SLC43A2, ↑SNX22, ↓TNFRSF21, TNRC4, VPS37C, WNK2, ↓ZNF367	Cellular Assembly and Organization, Nervous System Development and Function, Cell-To-Cell Signaling and Interaction	22	15
7	Actin, ↑ADCY7, ↑AKAP13, Calmodulin, ↑CDKN1A, ↑CLCN3, ↑COTL1, Cyclin A, ↓CYFIP2, F Actin, FSH, GHRL, HCRTR1, Histone h4, Insulin, ↑KCNQ1, ↑LRP1, ↓NPY, NPY1R, NPY2R, Pka, ↑PLEC1, PMCH, ↓PPP1R9A, ↑PSAP, PYY, Ras homolog, ↓RASAL2, Rb, RNA polymerase II, SMARCA4, STMN1, TTK, TYMS, ↓UNC13B	Connective Tissue Development and Function, Tissue Morphology, Behavior	21	14

8	Ap1, C5, Calpain, CaMKII, ↑CCL4, Creb, ERK1/2, ↓FAAH, ↑FOSL2, hCG, ↑HCK, Histone h3, Igm, IL12, Jnk, LAMB2, ↓LAMC1, Laminin, Mapk, ↓MME, Mmp, P38 MAPK, PDGF BB, PI3K, ↑PIK3R5, Pkc(s), PP2A, ↓SLC12A2, ↑SLC8A1, Smad, Tgf beta, ↑TNFRSF1B, ↑VCAN, Vegf, ↓ZBTB10	Ophthalmic Disease, Cellular Movement, Neurological Disease	20	14
9	↓AASS, ↓ABCB7, ABL1, ↓ACSM3, ASNS, CCNE2, CSTB, ↑CTSH, ↑DPYD, EIF4A1, EIF4E, FNG, IFI30, ↓ITM2C, JAK1, ↑JAKMIP1, ↑LFNG, MSH6, MYC, ↓MYO5C, NOP5/NOP58, NOTCH1, NOTCH2, ↑PHLDA2, ↓PLS3, RBL2, RBMS1, SERTAD1, SFTPB, ↓SLC39A8, SMARCA4, SMC4, STMN1, TGFB1, TYMS	Cancer, Cellular Growth and Proliferation, Cell Cycle	19	13
10	ACOT1, ↓AIF1L, BARD1, BAZ1B, BRIP1, ↓CEP290, CSF1, ↓DYNC2H1, DYNC2LI1, ↑ETV3, GHRH, HNF4A, HSD17B4, HUNK, ↓MGC29506, MLH1, MSH3, MSH6, ↓MYO1D, ↑MYO1G, ↓NEIL1, PCNA, POLB, ↓POU2AF1, progesterone, ↓PSD3, RBP1, ↓RIOK1, RIPK3, S100G, SMC1A, SPP1, ↑TSPAN14, ↓UQCC, XRCC1	DNA Replication, Recombination, and Repair, Gene Expression, Cell Cycle	19	13
11	3 BETA HSD, ABCD2, ↑AOAH, ARPP-21, ↓COBL, ↑DDX3Y, ↑EHBP1L1, ↑FAM65B, GHRL, HMGN3, HTT, IFITM1, IFNB1, L-triiodothyronine, ↑LMCD1, LTBP2, MFGE8, MGP, MYBPH, ↓NPY, PACSIN1, ↓PDZD2, ↓ROBO1, SEPP1, ↑SLC16A5, SLC27A1, SLC2A4, STMN1, TNF, TNNC1, ↓TPP2, TRIP10, TUBB, UCP1, UCP3	Carbohydrate Metabolism, Molecular Transport, Small Molecule Biochemistry	17	12
**In stromal cells of MDS-RARS (FDR <15% and fold≥1.7)**				
1	1,2-dipalmitoylphosphatidylcholine, ↑ALDH1A3, ALDH1B1, BCL2L14, CARD14, CASP8AP2, CBFA2T2, ↑CCL2, DNTTIP1, DOK5, ERK, ↑GRIA3, ↑HDAC9, IL1F9, LDL, ↑LIPG, MYEF2, NCOR1, Neuropilin, NFkB (complex), NR3C1, ↑NRP1, Orm, P38 MAPK, ↑PLA2G4A, PLXND1, ↑RDBP, RELT, Sema3, ↑SEMA3A, SEMA3B, SEMA3D, SEMA3E, ↓SPINT2, ↑TFPI	Cell Morphology, Cellular Compromise, Neurological Disease	27	11
2	ADCY9, ATF6, beta-estradiol, Ca2+, chondroitin sulfate B, CTSH, DEFB1, ↑EMR2, ESM1, FKBP7, ↑FKBP1A, GFM1, ↑GSTM3, HLA-DOA, ↑HLA-DPB1, ↓HLA-E, IFI30, IFNG, KDELR3, ↑KRT7, KRT13, KRT81, LOXL1, MAPK1, MBTPS, ↑MBTPS2, METAP2, PPIH, RYR3, ↑SEMA3A, ↑SOLH, TGFB1, ↑TNIK, TTC28, WISP1	Carbohydrate Metabolism, Cell Signaling, Molecular Transport	24	10

Genes (↑) up-regulated or (↓) down-regulated in MDS-RARS patients vs. healthy individuals.
