# Supplementary material for: Identification of protein-coding and non-coding RNA expression profiles in CD34+ and in stromal cells in refractory anemia with ringed sideroblasts
Source: BMC Med Genomics. 2010 Jul 15;3:30. doi: 10.1186/1755-8794-3-30 (PMC2914047; doi:10.1186/1755-8794-3-30)
Supplement: Additional file 3 — Transcripts with altered expression in stromal cells of MDS-RARS . This file can be viewed with: Adobe Acrobat Reader [file 1755-8794-3-30-S3.PDF]

Additional file 3. Transcripts with altered expression in stromal cells of MDS-RARS.

Gene Locus name[1]	Locus ID	Probe Coordinate	Probe Strand	Type	Orientation in relation to the protein coding gene	q value[2]	Fold Change
**down-regulated in MDS-RARS**							
SPINT2	10653	chr19:43474699-43474758	+	Exonic		0.000	-2.43
HLA-E	3133	chr6:30567339-30567398	+	Exonic		0.007	-1.73
**up-regulated in MDS-RARS**							
SEMA3A	10371	chr7:83235208-83235267	-	Exonic		0.021	4.42
SOLH	6650	chr16:529804-529859	+	Intronic	Sense	0.000	4.35
GRIA3	2892	chrX:122063785-122063829	+	Exonic		0.015	3.05
CROCC	9696	chr1:16968607-16968658	+	Intergenic	Sense	0.038	2.43
LEPREL1	55214	chr3:191157346-191157405	-	Exonic		0.019	2.41
TNIK	23043	chr3:172263055-172263114	-	Exonic		0.021	2.28
RGMB	285704	chr5:98159978-98160037	+	Exonic		0.044	2.02
TBCD	6904	chr17:78399392-78399451	+	Intronic	Sense	0.042	1.97
KRT7	3855	chr12:50917564-50917621	+	Exonic		0.039	1.91
SCFD2	152579	chr4:53581096-53581155	-	Exonic		0.000	1.84

[1] Gene locus name for intronic ncRNA is that of the protein-coding gene of the same locus; intergenic ncRNA is annotated with the name of the nearest protein-coding gene in that chromosome

[2] Minimum significance among all patient Leave-one-out analyses.
